# Combining Immune Checkpoint Inhibitors and Anti-Angiogenesis Approaches: Treatment of Advanced Non-Small Cell Lung Cancer

**DOI:** 10.3390/medsci13030143

**Published:** 2025-08-19

**Authors:** Tate Barney, Anita Thyagarajan, Ravi P. Sahu

**Affiliations:** 1 Boonshoft School of Medicine, Dayton, OH 45435, USA; barney.32@wright.edu; 2 Department of Pharmacology and Toxicology, Boonshoft School of Medicine, Dayton, OH 45435, USA; anita.thyagarajan@wright.edu

**Keywords:** non-small cell lung cancer, anti-angiogenesis, immunotherapy, bevacizumab, nivolumab

## Abstract

Combining immune checkpoint inhibitors (ICIs) and anti-angiogenic pharmacologic agents is an encouraging therapeutic approach in the treatment of non-small cell lung cancer (NSCLC). Currently, the only FDA-approved therapy combining an immune checkpoint inhibitor and a vascular endothelial growth factor (VEGF) inhibitor is atezolizumab, bevacizumab, and chemotherapy in first-line metastatic NSCLC patients. However, the combination of nivolumab, a programmed death-1 (PD-1) inhibitor, and bevacizumab has also shown encouraging results in patients with NSCLC with minimal adverse effects, respectively. This communication aims to highlight the efficacy of nivolumab and bevacizumab in NSCLC patients without sensitizing mutations in epidermal growth factor receptor (EGFR), anaplastic lymphoma kinase (ALK), or ROS proto-oncogene 1 (ROS1). In addition, the combination of nivolumab/atezolizumab and bevacizumab with other therapeutic agents is also discussed. We also underscore the adverse effects and limitations of such combinations in NSCLC patients. Future studies should focus on large-scale trials and biomarker identification to establish the benefits of these combination therapies in NSCLC patients.

## 1. Introduction

Lung cancer is one of the leading causes of cancer-related mortality worldwide and is the major cause of cancer deaths in the United States [1,2,3]. Non-small cell lung cancer (NSCLC) is the most common type of lung cancer, accounting for approximately 85% of all lung cancer cases [4,5]. Despite recent advancements in treatment modalities, the prognosis for patients with advanced or metastatic NSCLC without driver mutations remains poor [6,7]. Effective treatment options remain limited, especially for patients with resistance to standard therapies, but immunotherapy has changed the treatment of NSCLC, first in second-line treatment, then in first-line treatment, and now also in neoadjuvant and perioperative treatment [6,8,9]. This study is timely due to the lack of clinical trial data over the past 5 years discussing the combination of nivolumab and bevacizumab in NSCLC patients without sensitizing gene mutations. There is increasing clinical interest in identifying therapies to modulate tumor responses, but further studies of efficacy are warranted. The advent of immune checkpoint inhibitors (ICIs) targeting programmed death-1 (PD-1)/PD-ligand 1 (PD-L1) and cytotoxic T lymphocyte antigen 4 (CTLA-4) have revolutionized cancer therapy due to their ability to utilize the body’s own immune system to identify and destroy cancer cells [10,11,12,13]. However, ICIs face challenges due to immune evasion mechanisms within the tumor microenvironment (TME) [14,15]. ICIs foster a strong immune response, which enables the immune system, particularly T cells within TME, to better recognize and attack tumor cells. However, resistance to ICIs poses significant challenges to effective treatment options [14,15]. Given the critical role of angiogenesis in tumor survival and its impact on tumor progression, it has become a promising focus of research. Current efforts aim to overcome resistance to immunotherapy in lung cancer by targeting angiogenic pathways and exploring therapeutic strategies that combine immune checkpoint inhibitors (ICIs) with anti-angiogenic agents to enhance treatment efficacy and improve patient outcomes [16,17,18].

Notably, ICIs such as nivolumab, a monoclonal antibody targeting the PD-1 receptor, has demonstrated significant efficacy in NSCLC by disrupting the interaction of the PD-1 receptor with its ligands PD-L1 and PD-L2, thereby inhibiting the cellular immune response [19,20]. In addition, anti-angiogenic agents like bevacizumab, a targeted vascular endothelial growth factor (VEGF) inhibitor, have shown the ability to disrupt tumor vasculature, thereby inhibiting tumor growth and metastasis [21,22,23]. The idea for combining nivolumab with bevacizumab lies in their complementary mechanisms of action: while nivolumab stimulates T-cell-mediated immune responses, bevacizumab reduces angiogenesis, which can modify the TME to enhance immune cell infiltration and function [19,21,22,23]. This combination therapy has the potential to overcome resistance mechanisms that limit the efficacy of monotherapies, offering a more comprehensive approach to treating NSCLC. Clinical trials have provided encouraging results, showing improved PFS and OS in patients treated with the combination of nivolumab and bevacizumab, particularly when used alongside platinum-based chemotherapy [24,25,26,27]. The goal of this communication is to discuss the efficacy of targeting immune checkpoints and angiogenesis in patients without having sensitizing mutations in epidermal growth factor receptor (EGFR), anaplastic lymphoma kinase (ALK), or ROS proto-oncogene 1 (ROS1) oncogenes, and to highlight the exploration of combination therapy for NSCLC treatment without utilizing predictive biomarker responses.

## 2. Mechanisms of Action of Nivolumab and Bevacizumab

Nivolumab (OPDIVO) is an FDA-approved drug available as a single-use injection for a variety of human malignancies, including NSCLC, small cell lung cancer (SCLC), pleural mesothelioma, melanoma, squamous cell carcinoma of the head and neck, renal cell carcinoma, urothelial carcinoma, hepatocellular carcinoma, colorectal cancer, Hodgkin lymphoma, and esophageal squamous cell carcinoma [19,28]. Nivolumab is a human IgG4 monoclonal antibody checkpoint inhibitor that binds to programmed death-1 receptors (PD-1) expressed on activated T-cells. When PD-1 binds to its ligands, PD-L1 and PD-L2, which are expressed on antigen presenting cells (APCs) and other cells, inhibitory signals are sent to T-cells. This dampens the immune response, helping to maintain tolerance and prevent tissue damage from excessive immune activity [19]. However, abnormal PD-L1 expression by tumor cells utilize this pathway to evade proper immune detection [19]. When PD-L1 on tumor cells binds to PD-1 on T-cells, the lymphocytes become deactivated and allow the tumor cells to evade immune recognition and continue proliferation unchecked. Nivolumab blocks the interaction between PD-1 and PD-L1/PD-L2 to prevent the inhibitory signal from being transmitted, allowing T-cells to remain active and recognize and destroy tumor cells [19,29,30].

**Figure 1:**
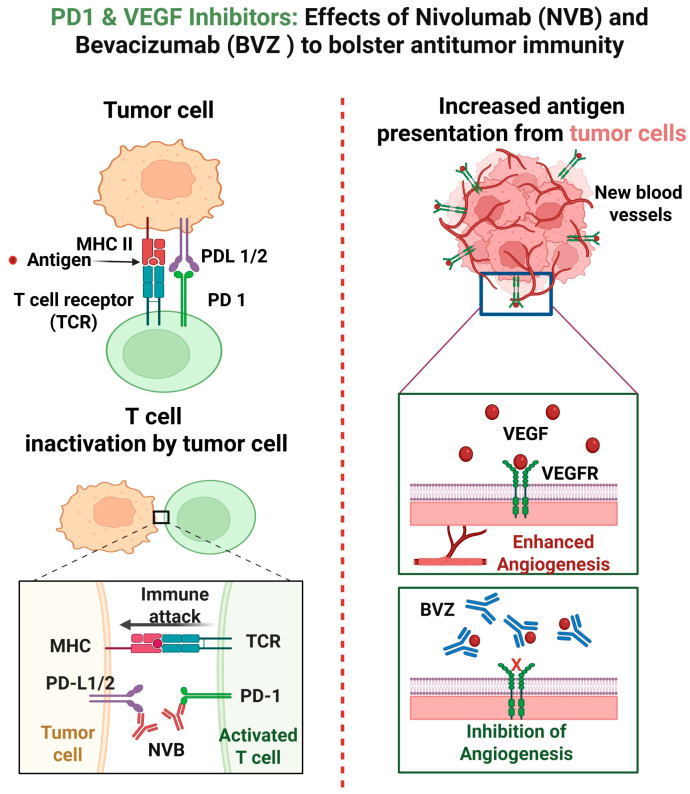
Mechanisms of action of nivolumab and bevacizumab.

Bevacizumab (AVASTIN) was first approved by the FDA in 2004 as an intravenous (IV) infusion and is currently approved for metastatic colorectal cancer, non-squamous NSCLC, glioblastoma, metastatic renal cell carcinoma, and persistent, recurrent, or metastatic carcinoma of the cervix [31]. Bevacizumab is a recombinant humanized monoclonal IgG1 antibody that binds to VEGF to inhibit its binding to the cell surface VEGF receptors for both the tumor cells as well as the local healthy cells [31]. This creates two different mechanisms by which bevacizumab can combat cancer cells. By blocking VEGF from binding to healthy endothelial cells, it can effectively block the signaling for angiogenesis and disrupt the creation of blood vessels needed by the tumor cells [17,21,22,32,33]. When VEGF is blocked from binding to the receptors directly on the tumor cells, it can inhibit the signaling for cell proliferation and survival. VEGF is also an important immunomodulator of TME. The mechanism of actions of nivolumab and bevacizumab is depicted in Figure 1.

When used in combination, nivolumab and bevacizumab offer complimentary mechanisms. Despite this synergy, resistance to the combination may still arise. VEGF inhibitors can lead to hypoxia driven upregulation of other pro-angiogenic pathways. Similarly, not all tumors respond to PD-1 blockage due to antigen presentation defects.

## 3. Mechanism of Resistance and Adverse Reactions of Nivolumab and Bevacizumab

Patients treated with immune checkpoint therapy such as nivolumab can acquire resistance to the drug through mechanisms that are not very well understood [27]. Many theorize that resistance could be multifactorial, including ideas such as the TME including various factors and cell types such as regulatory T cells (Tregs), myeloid-derived suppressive cells (MDSCs), tumor-associated macrophages (TAMs), and immature dendritic cells (imDC) [18]. Low or variable PD-L1 expression in NSCLC tumors and other associated mechanisms might not respond well to nivolumab [27]. Certain mutations in genes such as rearranged during transfection (RET) or human epidermal growth factor receptor 2 (HER2) can also reduce the expression of PD-L1, thus reducing the effectiveness of nivolumab [27]. Additionally, low tumor mutation burden (TMB) can increase the likelihood of nivolumab and other PD-1 inhibitors being an ineffective treatment option because these tumors are unable to elicit a strong immune response to PD-1 inhibiting drugs [10,34]. Other mechanisms of resistance involve insufficient antigen recognition by T-cells and other downstream effects of issues in the T-cell activation process [35]. Additional factors that affect the resistance of ICIs such as nivolumab include EGFR mutations and ALK alterations [36]. Moreover, associated adverse reactions are other potential challenges to effective treatments. The FDA cites several adverse reactions to nivolumab as a single agent as well as in combination with ipilimumab, platinum chemotherapy, cabozantinib and with fluoropyrimidine [28]. For example, nivolumab can cause severe infusion-related reactions, which have been reported in <1.0% of patients in clinical trials [28]. Fatigue, musculoskeletal pain, diarrhea, nausea, rash, pruritus, asthenia, cough, dyspnea, constipation, decreased appetite, back pain, upper respiratory tract infection, arthralgia, pyrexia, headache, abdominal pain, vomiting, and urinary tract infection are other adverse effects of nivolumab [28].

While bevacizumab impacts tumor proliferation in multiple ways, patients receiving bevacizumab have shown acquired resistance to the treatment [32]. The specific mechanisms of resistance of bevacizumab are still being studied; however, there are several factors that are thought to play a role in the resistance [32,33]. First, while VEGF is a powerful angiogenic signaling molecule, it is not the only signal for angiogenesis, and it is thought that the tumor cells can upregulate other angiogenic signaling pathways, such as hypoxia-inducible factor-1 alpha (HIF-1α) in the presence of bevacizumab [32]. Second, the TME seems to broadly impact the efficacy of chemotherapeutic agents and bevacizumab. It seems hypoxia is the most significant factor in the TME, which is consequently increased by blocking angiogenesis with a VEGF inhibitor [32,35,37]. In fact, it is documented that the hypoxia induced by an angiogenesis inhibitor has the potential to worsen tumor invasiveness and metastasis [37]. Notably, there are several implications of hypoxia in the TME that can lead to tumor survival, but among them is the hypoxia’s recruitment of myeloid cells into the TME that ultimately facilitate an immunosuppressive microenvironment and a weakened antitumor response [32]. Moreover, induction of hypoxic condition mediated through VEGF inhibitors reduces the recruitment of suppressive cells into the TME to improve the efficacy of immunotherapies [18,27]. Additionally, adverse reactions to bevacizumab are also potential challenges to effective treatment. Bevacizumab is also associated with several adverse reactions that occurred in greater than 10% of patients taking the medication. These adverse reactions include epistaxis, headache, hypertension, rhinitis, proteinuria, taste alteration, dry skin, rectal hemorrhage, lacrimation disorder, back pain, and exfoliative dermatitis [31]. According to the FDA, bevacizumab was discontinued in 8.4–21% of patients across all studies because of these reactions. Of note, the maximum dose tested in humans was 20 mg/kg IV, and nine of the sixteen patients developed a headache, three of which experienced severe headaches [31].

## 4. Studies of Nivolumab and Bevacizumab Combination with or Without Other Agents

A focused literature search was conducted using PubMed for articles published between 2020 and 2024. Keywords included ‘non-small cell lung cancer,’ ‘nivolumab,’ ‘bevacizumab,’ ‘immune checkpoint inhibitors,’ and ‘anti-angiogenesis.’ Only English-language articles and clinical trials involving human subjects were included, yielding the four studies discussed below. The two drugs, nivolumab and bevacizumab, have been studied in clinical trials in addition to therapies such as platinum chemotherapy, cell cycle inhibitor chemotherapy, and DNA synthesis inhibitor chemotherapy [24,25,26,38]. There is a gap in our knowledge of how nivolumab and bevacizumab combination without additional chemotherapy interact as a potential therapy for treatment in NSCLC. The summary of clinical studies with a combination of nivolumab and bevacizumab is given in Table 1.

One clinical trial assessed the approach of combining nivolumab or placebo with bevacizumab plus platinum-based chemotherapy for treatment of NSCLC patients without driver mutations in ALK, EGFR, or ROS1, evaluated the safety and effectiveness in the treatment plan [24]. The 550 patients were randomized. At the time of the analysis (minimum follow-up: 19.4 months), the median overall survival (OS) was longer in the nivolumab arm than in the placebo arm (30.8 vs. 24.7 months; hazard ratio 0.74; 95% confidence interval 0.58–0.94). The 12-month OS rates were 81.3% vs. 76.3% in the nivolumab vs. placebo arms, respectively. The respective 18-month OS rates were 69.0% vs. 61.9%. This treatment regimen was identical to their previous clinical trial and concluded that nivolumab in combination with platinum chemotherapy and bevacizumab demonstrated a longer OS compared to the placebo combination [24]. This study did not mention adverse events in their conclusion.

In an additional study, nivolumab was assessed with carboplatin, paclitaxel, and bevacizumab in NSCLC patients with stage IIIB/IV or recurrent NSCLC without sensitizing EGFR, ALK, or ROS1 alterations [25]. This study initially used carboplatin, paclitaxel, and bevacizumab every 3 weeks for up to six cycles and had maintenance therapy using nivolumab/placebo with bevacizumab [25]. This study included a cohort of 550 patients, 273 received the nivolumab and 275 received placebo combinations, respectively. With a median follow up of 13.7 months, the Independent Regulatory Review (IRRC)-assessed that the median progression free survival (PFS) was significantly longer in the nivolumab arm than in the placebo arm (12.1 versus 8.1 months; hazard ratio 0.56; 96.4% confidence interval 0.43–0.71). The PFS benefit was observed across all patients with any PD-L1 expression levels, including PD-L1-negative patients. This study concluded that nivolumab/bevacizumab with cytotoxic chemotherapy should be considered as it demonstrated significant improvement in PFS [25]. The incidence of treatment related adverse events was comparable between the control and treatment groups. Treatment related events leading to death were observed in five and four patients in the treatment and placebo group, respectively [25].

An additional clinical trial assessed nivolumab efficacy in different combinations of chemotherapeutic agents for NSCLC without targetable oncogenes, respectively [26]. The patients were divided into four treatment arms. Arm A includes four cycles of cisplatin and gemcitabine; arm B includes four cycles of cisplatin and pemetrexed followed by pemetrexed maintenance therapy; arm C includes four to six cycles of carboplatin, paclitaxel, and bevacizumab followed by bevacizumab; and arm D includes docetaxel. In this trial, minimum follow-up period was 57.9 months. Median PFS was 6.3 (0.7+–47.8), 11.8 (1.4–65.1+), 40.7 (5.3–60.8+), and 3.2 (1.9–10.9) months, and 5-year PFS was observed in 0/6, 1/6, 1/6, and 0/6 patients in arms A, B, C, and D, respectively. Median OS was 13.2 (11.0–55.4), 28.5 (14.6–66.2+), not reached (24.2–67.4+), and 12.5 (9.8–16.9) months, and the number of patients surviving 5 years were 0/6, 1/6, 4/6, and 0/6 in arms A, B, C, and D, respectively. This trial concluded that the nivolumab/carboplatin/paclitaxel/bevacizumab therapy combination showed both tolerability and 5-year PFS and OS [26]. No unexpected severe adverse events or treatment-related deaths occurred in this trial [26].

A systematic review assessed checkpoint inhibitor combinations, including pembrolizumab, atezolizumab, and atezolizumab/bevacizumab for NSCLC treatment. This review showed significantly improved OS compared with controls in patients with advanced NSCLC without EGFR/ALK mutations [38]. Two trials reported outcomes for squamous NSCLC, with pembrolizumab–chemotherapy reporting significantly improved (OS) compared with chemotherapy. Of note, the combination of nivolumab–ipilimumab failed to improve OS [38]. Outcomes for atezolizumab–bevacizumab–chemotherapy in EGFR+/ALK+ patients are promising and require further exploration. The results of this review indicated that pembrolizumab (PD-1 inhibitor) improved OS, and the atezolizumab/bevacizumab combination therapy for EGFR+/ALK+ patients requires additional studies [38].

While these studies collectively demonstrate the potential of combining ICIs and anti-angiogenesis agents to benefit patients with NSCLC, there are important limitations and discrepancies. Variations in trial design, chemotherapy regimens, follow-up periods, and reporting of adverse events limit direct comparison. This highlights the need for standardized study designs and biomarker-driven strategies in future studies.

## 5. Potential of Other ICIs and Anti-Angiogenic Therapies for NSCLC

In addition to the combination of nivolumab and bevacizumab, several other ICIs have been studied in combination with anti-angiogenic therapies for the treatment of NSCLC [18,39]. The other considerable therapies are listed in Table 2.

Atezolizumab, an anti-PD-L1 antibody, has been evaluated in combination with bevacizumab in a phase III clinical trial [18]. This study demonstrated that the combination, along with chemotherapy, improved PFS and OS in patients with metastatic non-squamous NSCLC [18]. The median PFS of the group in combination with atezolizumab, carboplatin, paclitaxel, and bevacizumab was 8.3 months, and the carboplatin, paclitaxel, and bevacizumab group PFS was 6.8 months (HR: 0.59). The median OS was 19.2 months for the first group, and 14.7 months for the second 31 group (HR: 0.78) [18]. The anti-angiogenic effects of bevacizumab are thought to enhance the immune response elicited by atezolizumab. The incidence of treatment-related adverse events was 25.4% for the atezolizumab, carboplatin, paclitaxel, and bevacizumab group and 19.3% for the bevacizumab, carboplatin, and paclitaxel group. However, 77.4% of atezolizumab, bevacizumab, carboplatin, and paclitaxel (ABCP) patients had grade 1–2 AEs [18].

Pembrolizumab has been combined with lenvatinib, a tyrosine kinase inhibitor (TKI) that includes anti-angiogenic properties such as targeting VEGF and fibroblast growth factor (FGF) [39]. This combination has shown potential in enhancing anti-tumor activity by modulating the TME [39]. In addition, preclinical studies suggest that lenvatinib can decrease TAMs and Tregs, thereby improving the efficacy of ICIs [39,40].

Pembrolizumab has also been combined with ramucirumab, an anti-VEGFR-2 antibody [40]. An open-label phase 1a/b trial found median PFS at 9.3 months, while 12-month and 18-month PFS were each 45%. Median OS was not reached, but the 12-month and 18-month OS rates were 73% and 64%, respectively [40]. The rationale is that inhibiting VEGFR-2 can normalize tumor vasculature and reduce immunosuppressive cells, thereby potentiating the effects of pembrolizumab [40]. Adverse effects associated with this therapy were fatigue and myocardial infarction in 7% of 31 patients [18,40].

## 6. Conclusions and Future Perspectives

NSCLC has a variety of treatment options for patients lacking EGFR, ALK, or ROS1 mutations. The data demonstrated that nivolumab in combination with bevacizumab and platinum-based chemotherapy provide promising results in improving PFS and OS in patients with NSCLC. While few clinical trials demonstrated the beneficial effects of these immunotherapies, further research is needed to assess the efficacy of combination immunotherapy in patients lacking gene mutations. In addition, while biomarkers aid in the diagnosis of disease pathology, identifying biomarkers that predict response to combination therapy remains a challenge. Moreover, lack of accuracy in biomarkers can lead to unnecessary pharmacological interventions and potential adverse effects which should be monitored carefully in patients receiving treatment. While biomarkers such as PD-L1 expression are useful in predicting nivolumab efficacy, they are not reliable predictors of adverse events. Although nivolumab generally has a favorable safety profile, further research is needed to identify biomarkers that can guide both therapeutic response and toxicity risk. Bevacizumab has been reported to have adverse reactions in greater than 10% of patients. Taken together, nivolumab and bevacizumab should be evaluated with careful consideration with other therapeutic agents for the treatment of NSCLC. Additionally, current clinical data on ICIs and anti-angiogenesis combinations in NSCLC are currently limited by small sample sizes and short follow-up periods and should aim to incorporate more diverse patient populations. Emphasis should be placed on longitudinal sampling to monitor changes in the TME and immune system functionality. Future research should focus on developing more comprehensive biomarker panels that combine immune markers, angiogenesis markers, and characteristics of the TME. Gaining a better understanding of resistance mechanisms could help inform the design of combination therapies and improve how treatments are timed to extend patient response.

## Figures and Tables

**Table 1:** Evidence of nivolumab and bevacizumab studies with or without other therapeutic agents for NSCLC is summarized.

Patient Population	Study Design	Intervention	Control	Primary Endpoint	Key Results	Refs.
Treatment- naïve stage HIB/IV NSCLC	Randomized 1:1 trial	Nivolumab with bevacizumab + platinum- based chemo	Placebo + chemo	OS	OS: 30.8 vs. 24.7 months (HR 0.74) CI: 0.58–0.94	[24]
Treatment- naïve stage IIIB/IV NSCLC	Randomized, double- blind, 1:1 trial	Nivolumab with bevacizumab, paclitaxel + platinum-based chemo	Placebo + chemo	PFS	PFS: 12.1 vs. 8.1 months (HR 0.56) CI: 0.43–0.71	[25]
Japanese patients with NSCLC	Phase Ib	Nivolumab with bevacizumab, paclitaxel + platinum-based chemo	None	PFS and OS	PFS: 40.7 months, OS: 28.5 months CI: not reported	[26]
Advanced NSCLC	Meta- analysis of phase III clinical trials	Nivolumab + ipilimumab with bevacizumab	Chemo alone	OS	Improved OS in EGFR/ ALK mutated patients CI: not reported	[38]

**Table 2:** Evidence from other ICI and anti-angiogenic-based approaches for NSCLC is summarized.

Patient Population	Study Design	Intervention	Control	Primary Endpoint	Key Results	Refs.
Metastatic non-squamous NSCLC	Phase III clinical trial	Bevacizumab + Carboplatin + Paclitaxel	Atezolizumab + Bevacizumab + Carboplatin + Paclitaxel	PFS and OS	Median PFS: 8.3 months (Intervention) vs. 6.8 months (Control) (HR: 0.59, *p* < 0.0001); Median OS: 19.2 months (Intervention) vs. 14.7 months (Control) (HR: 0.78, *p* = 0.02)	[18]
Metastatic NSCLC	Preclinical and clinical studies	Pembrolizumab + Lenvatinib	Not specified	Modulation of TME	Lenvatinib may decrease TAMs and Tregs, improving ICI efficacy (*p* < 0.01)	[39]
Metastatic NSCLC	Open-label phase la/b trial	Pembrolizumab + Ramucirumab	Not specified	PFS and OS	Median PFS: 9.3 months; 12-month and 18-month PFS: 45%; 12-month and 18-month OS: 73% and 64% Respectively (*p* value not reported)	[40]

## Data Availability

No new data were created or analyzed in this study.

## Abbreviations

NSCLC	Non-small cell lung cancer
ICIs	Immune checkpoint inhibitors
PFS	Progression free survival
OS	Overall survival
EGFR	Epidermal growth factor receptor
ALK	Anaplastic lymphoma kinase
ROS1	ROS proto-oncogene 1
PD-1	Programmed death-1
PD-L1	PD-ligand 1
CTLA-4	cytotoxic T lymphocyte antigen 4
TME	Tumor microenvironment
VEGF	Vascular endothelial growth factor
APC	Antigen presenting cells
Tregs	Regulatory T cells
MDSCs	Myeloid-derived suppressive cells
TAMs	Tumor-associated macrophages
imDC	Immature dendritic cells
RET	Rearranged during transfection
TMB	Tumor mutation burden
HIF-1α	Hypoxia-inducible factor-1 alpha
IRRC	Independent Regulatory Review
FGF	Fibroblast growth factor
TKI	Tyrosine kinase inhibitor
